# Editorial: Molecular evolution: You learn from your mistakes

**DOI:** 10.3389/fmolb.2022.985289

**Published:** 2022-08-17

**Authors:** Marco Fantini, Edoardo Sarti, Gian Gaetano Tartaglia, Annalisa Pastore

**Affiliations:** ^1^ BioSNS Laboratory of Biology, Scuola Normale Superiore (SNS), Pisa, Italy; ^2^ Algorithms, Biology, Structure (ABS), Inria at Université Côte d’Azur, Valbonne, France; ^3^ Centre for Genomic Regulation (CRG), The Barcelona Institute for Science and Technology, Barcelona, Spain; ^4^ RNA System Biology Lab, Centre for Human Technologies, Istituto Italiano di Tecnologia (IIT), Genoa, Italy; ^5^ Centre for Genomic Regulation (CRG) and ICREA, The Barcelona Institute for Science and Technology, Barcelona, Spain; ^6^ Dipartimento di Biologia e Biotecnologie, Sapienza University, Rome, Italy; ^7^ UK-DRI Centre at the Maurice Wohl Institute, Department of Clinical and Basic Neuroscience, King’s College London, London, United Kingdom

**Keywords:** molecular evolution, mutagenesis, *in vitro* evolution, evolutionary biology, structural biology, mutational data analysis

What is the order of magnitude for evolutionary processes to be observed and which are the tools that enable us to study the viable paths in mutational evolutionary landscape?

Evolution is a phenomenon traditionally studied at different scales of magnitude. In the last century, the community put a great emphasis in connecting the results obtained at different scales to build our current understanding of the subject. The building blocks of this knowledge are experiments executed at the smallest molecular scale to prove fundamental concepts. These concepts are then connected and interweaved with previous knowledge to perfect the current models of evolution.

In this Research Topic we explored the process of generating molecular evolution where artificial mutagenic pressure is used to create a diverse library of mutants and the tools used to generate and analyze such diversity.

In this Research Topic, Arthur M. Lesk gives his perspective on the methods to determine the three-dimensional structure using correlated mutations emerged during evolution (Marks et al., 2011). The article gives us both a historical perspective on these methods and the author’s perspective of new ways to artificially generate the evolutionary constraints that power these algorithms.

In a related article, Monti et al. use Zyggregator (Tartaglia et al., 2008), an algorithm that estimates whether a protein aggregates into solid-like aggregates based on combinations of physico-chemical properties such as hydrophobicity, helical and beta-propensities, to investigate the role of protein aggregation in evolution. By analyzing two mutagenic datasets of proteins (Bolognesi et al., 2019; Fantini et al., 2019) for which aggregates have opposite effects in the likelihood of cell survival, they demonstrate how different selective pressures drastically change the effect of aggregation on a specific system: an exogenous protein with no functional role, such as TDP-43 in *S. cerevisiae* is less toxic if compartmentalized in an aggregate, while an endogenous proteins, such as TEM-1 beta lactamase in *E. coli*, is functional when aggregation is avoided.

Similarly using the TEM ß-lactamase model, Alejaldre et al. focus on a different aspect of protein evolution: catalytic speed and substrate specificity. Despite showing vastly differing patterns of protein dynamics at the timescale of catalytic turnover (Gobeil et al., 2019), all their models demonstrate to possess the high evolvability of TEM-1 ß-lactamases. Loop movements and active-site cavity fluctuations occur at slow timescales and are relevant for catalysis, in contrast fast timescales are associated with the formation and breakdown of transition state. The tolerance to extensive protein dynamics at slow timescales is consistent with the robustness of TEM-1 β-lactamases and has been hypothesized to facilitate evolution towards the recognition of new substrates. Dynamics at fast timescales are instead largely conserved in the three β-lactamase hosts used in this study. This backdrop of conserved fast motions and diverse slow motions provides scaffolds that have the potential to evolve toward new protein function.

Finally, in a review influenced by the recent SARS-CoV-2 pandemic and previous epidemic emergencies, Narayanan and Procko analyze and compare the way deep mutational scanning or deep mutagenesis have been influencing the study of the interaction between viral glycoprotein and their host receptors (Haddox et al., 2016; Chan et al., 2020). The authors conclude that these techniques, whether used with live viral libraries passed through cell culture, or expression of protein variants by yeast surface display or in mammalian cells, allow for the residue-level mapping of functional interaction sites, structural modeling, and prediction of escape mutations in response to selective pressures such as antibodies, small molecule drugs, and other therapeutics.

Taken together these articles provide a snapshot of the current Research on evolution at the molecular level, showing the relationship between evolution and physico-chemical properties of proteins and their dynamics. Most importantly, these works generate information that link protein folding and interactions.

We hope that the articles collected in this Research Topic are able to convey the multifaceted nature of evolution at the molecular level and would be valuable to future scholars to understand the multiple layers of information that can be generated by mutagenesis.
